# Antiretroviral therapy and liver disease progression in HIV and hepatitis C co-infected patients: a systematic review and meta-analysis

**DOI:** 10.1186/s41124-016-0015-7

**Published:** 2016-08-15

**Authors:** Alexis Llewellyn, Mark Simmonds, Will L Irving, Ginny Brunton, Amanda J Sowden

**Affiliations:** 1 Centre for Reviews and Dissemination, University of York, York, UK; 2 UCL Institute of Education, University of London, London, UK; 3 Faculty of Medicine & Health Sciences, University of Nottingham, Nottingham, UK

**Keywords:** Systematic review, Meta-analysis, Anti-retroviral agents, Hepatitis C, HIV

## Abstract

**Background:**

HIV co-infection exacerbates hepatitis C disease, increasing the risk of cirrhosis and hepatitis C-related mortality. Combination antiretroviral therapy (cART) is the current standard treatment for co-infected individuals, but the impact of cART and antiretroviral (ARV) monotherapy on liver disease in this population is unclear. We aimed to assess the effect of cART and ARV monotherapy on liver disease progression and liver-related mortality in individuals co-infected with HIV and chronic hepatitis C.

**Methods:**

A systematic review with meta-analyses was conducted. MEDLINE and EMBASE bibliographic databases were searched up to September 2015. Study quality was assessed using a modified Newcastle-Ottawa scale. Results were synthesised narratively and by meta-analysis.

**Results:**

Fourteen observational studies were included. In analyses that adjusted for potential confounders, risk of liver-related mortality was significantly lower in patients receiving cART (hazard ratio/odds ratio 0.31, 95 % CI 0.14 to 0.70). Results were similar in unadjusted analyses (relative risk 0.40, 95 % CI 0.29 to 0.55). For outcomes where meta-analysis could not be performed, results were less consistent. Some studies found cART was associated with lower incidence of, or slower progression of liver disease, fibrosis and cirrhosis, while others showed no evidence of benefit. We found no evidence of liver-related harm from cART or ARV monotherapy compared with no HIV therapy.

**Conclusions:**

cART was associated with significantly lower liver-related mortality in patients co-infected with HIV and HCV. Evidence of a positive association between cART and/or ARV monotherapy and liver-disease progression was less clear, but there was no evidence to suggest that the absence of antiretroviral therapy was preferable.

**Electronic supplementary material:**

The online version of this article (doi:10.1186/s41124-016-0015-7) contains supplementary material, which is available to authorized users.

## Background

Hepatitis C is an infectious liver disease caused by the hepatitis C virus (HCV). Hepatitis C infections occur if the virus is able to enter the blood stream and reach the liver. Co-infection with hepatitis C virus (HCV) and human immunodeficiency virus (HIV) is common due to similar modes of transmission. There are an estimated 7 million individuals worldwide co-infected with HCV and HIV [1]. Chronic HCV infection affects approximately 6.2 % of HIV positive individuals, with greater rates in intravenous drug users [2]. HIV co-infection exacerbates HCV disease, increasing the risk of cirrhosis and HCV-related mortality [3].

In high-income countries, the widespread use of monotherapy with an antiretroviral drug (or ARV monotherapy) in the late 80s, followed by combination antiretroviral therapy (cART) since 1996 has resulted in HIV-infected patients living longer, and chronic HCV infection is now the second most common cause of death, after AIDS-related complications, among HIV-infected individuals in areas where cART is available [1]. The effect of cART on the clinical course of HCV infection is not clear. It has been suggested that the HIV viral suppression [4] and immune reconstitution possible with cART are critical factors that slow down the rate of HCV fibrosis progression [5]. However, some studies have reported that cART may adversely affect hepatitis C-related outcomes by increasing HCV viral load, liver toxicity and fibrosis progression [6–8].

Today, most individuals infected with HCV in high-income countries acquire the virus through unsterile drug injecting practices. However, before the introduction of effective blood donor screening, individuals became infected through blood transfusion or therapy with medical products manufactured from donated human blood. It is estimated that blood transfusion resulted in approximately 23,500 HCV transmissions during the 1970s and 1980s in England, [9] and around 28,000 in the UK, [10] before an effective blood donor screening test was introduced in the UK in 1991. More than 4,600 patients with bleeding disorders were also infected via treatment with HCV-contaminated plasma products. Since 2004, those surviving patients who acquired chronic HCV infection through NHS contaminated blood or blood products before donor screening tests or virus inactivation methods were available have received financial help via a UK wide ex-gratia scheme established by the Department of Health [11].

We report the findings from a systematic review that was commissioned by the Department of Health, England [12]. cART is the current standard treatment for this patient group but its impact on liver disease progression and liver related mortality is unclear. Evidence of harm associated with cART and/or ARV monotherapy may have implications for compensation policies for people who acquired HCV through contaminated blood products prior to 1991. The findings from an earlier review examining the association between cART and ARV monotherapy and liver disease outcomes were inconclusive [13]. Since publication of the review in 2007 new primary studies have become available and an up-to-date review of the available evidence is needed.

## Methods

We followed the general principles recommended in Centre for Reviews and Dissemination (CRD) Guidance for Undertaking Reviews in Health Care, [14] and the reporting guidance of the PRISMA and MOOSE statements [15, 16].

### Search strategy

MEDLINE and EMBASE electronic databases were searched up to September 2015 for studies published in English. We applied no date restrictions or study design filters. Search terms included “hepatitis C”, “HIV”, “antiretroviral therapy”, and “liver disease”. The reference lists of relevant published reviews were checked for additional studies [13, 17–19]. A full search strategy is reported in the Additional file 1.

### Study selection

Studies evaluating the effect of cART and/or ARV monotherapy in individuals co-infected with HIV and HCV were eligible for inclusion. Studies had to include a comparison group of participants who did not receive the intervention. Studies that measured treatment exposure and outcome at the same point in time were excluded because they were not considered suitable for measuring disease progression.

The two outcomes of interest were liver-related mortality and liver disease progression, and the latter includes progression to/of fibrosis and cirrhosis; decompensated liver disease; end-stage liver disease; and hepatocellular carcinoma. Outcomes had to be measured using liver biopsy or a validated non-invasive method. Studies examining HCV viral load or transaminase/aminotransferase only were excluded. Data had to be presented as, or allow calculation of, relative risks (RR), odds ratios (OR), hazard ratios (HR), or mean differences (MD).

Titles and abstracts were screened by a single reviewer, and full papers were assessed by two reviewers independently, with disagreements resolved through discussion.

### Data extraction and risk of bias

Relevant study details and patient characteristics (e.g., age, sex, baseline liver disease severity, mode of HCV/HIV infection; HIV/HCV treatment regimens and history; concomitant treatments) and outcomes were extracted into standardised forms. Where outcomes were reported with different levels of adjustment (e.g., adjusting for age and sex only versus age, sex and time-dependent covariates), data with the greatest number of adjustments were preferred. Risk of bias was evaluated using a modified version of the Newcastle-Ottawa quality assessment tool [20]. Three main domains were considered: participant selection, confounding, and outcome measurement. Further details are reported in the Supporting Information. Data were extracted by a single reviewer and checked by a second, with disagreements resolved through discussion. Where relevant, study authors were contacted for missing data.

### Synthesis

Results for liver-related mortality and liver disease progression were pooled in a meta-analysis if at least two studies reported that outcome, and if data were reported consistently enough for analysis to be feasible. Otherwise, results were synthesised narratively. Where meta-analyses were performed, studies were pooled using standard random-effects DerSimonian-Laird meta-analyses [21]. Heterogeneity was assessed through visual inspection of forest plots and using I^2^ [22]. When pooling adjusted odds, hazard or risk ratios these were assumed to be equivalent regardless of the specific statistic reported or which covariates were used in adjusted models. Adjusted and unadjusted ratios were pooled using the inverse variance method. Meta-analyses were conducted using R software.

Where participants from several studies were recruited from the same cohorts and significant overlap was suspected, data from only one study with the most reliable reporting were included in the main analyses. The impact of suspected overlap in participants across studies was explored in sensitivity analyses, as was the use of composite outcomes (one study reported end-stage liver disease, hepatocellular carcinoma or death only as a composite outcome [23]).

Where possible, pre-planned subgroup analyses including only studies with a large proportion of patients with haemophilia were conducted. Meta-regression analyses or other subgroup analyses were considered inappropriate due to the small number of studies.

## Results

**Fig. 1:**
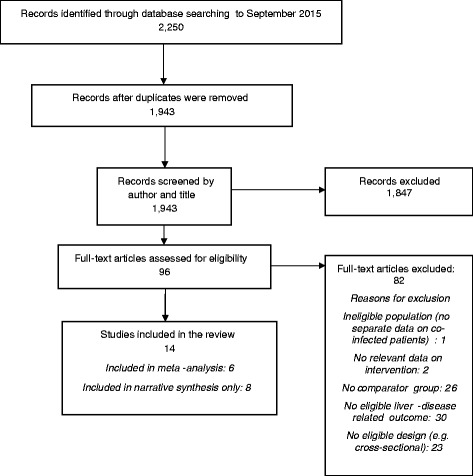
Flow of studies

The bibliographic searches yielded a total of 1,943 unique records. From these, 96 studies of potential relevance were identified and 14 studies met our inclusion criteria (see Fig. 1 for further details).

### Risk of bias

As would be expected in observational studies, risk of confounding of results due to unmeasured factors was the most frequent quality concern in the included studies, with ten studies classed at moderate or high risk of bias. Risk of bias associated with participant selection was considered unclear due to limited reporting in five studies, and low in nine studies (where the study sample was considered broadly representative of the population of interest, and assessment of HIV/HCV and outcome at baseline were considered appropriate). The risk of bias associated with outcome measurement was considered mostly low, as ten studies measured and reported their outcomes using appropriate methods. Further details about quality criteria and judgments are reported in Table 1 and Additional file 2.

**Table 1:** Risk of bias

	Selection bias	Confounding bias	Outcome measurement bias
Bruno (2007) [34]	Low	Moderate	Low
Giron-Gonzalez (2007) [29]	Unclear	Moderate	Low
Limketkai (2012) [23]	Low	Low	Low
Macias (2006) [25]	Low	Moderate	Low
Macias (2009) [24]	Low	Low	Unclear
Mariné-Barjoan (2004) [35]	Low	High	Low
Mehta (2005) [30]	Unclear	High	High
Merchante (2006) [31]	Unclear	High	Low
Pineda (2009) [32]	Low	Moderate	Low
Qurishi (2003) [26]	Unclear	Low	Low
Ragni (2009) [27]	Low	Low	Unclear
Reiberger (2010) [36]	Low	High	Low
Sanmartin (2014) [33]	Low	High	High
Schiavini (2006) [28]	Unclear	High	Low
Total risk of bias	9 low	4 low	10 low
	0 high	6 high	2 high
	5 unclear	4 moderate	2 unclear

### Study characteristics

Most studies were carried out in Europe, with six from Spain, two from Italy and one in each of France, Germany and Austria. Three studies were carried out in the USA. Study dates ranged from 1970 to 2011 and six studies were conducted across the pre-post cART era (before and after 1996) [23–28]. Seven studies followed patients prospectively in time [23, 27, 29–33] and the remaining seven studies were classed as retrospective [24–26, 28, 34–36].

Many participants were current or past injection drug users (IDU), with eight studies having IDU rates of 72 % or above. One study focused on patients with haemophilia exclusively [27], and another reported that 81 % of patients had the condition [26]. The other studies failed to report the number of patients with haemophilia. Baseline liver damage severity varied across the studies: ten studies included no or few patients with cirrhosis [23–28, 30, 33, 35, 36]; four studies included only patients with compensated cirrhosis at baseline [29, 31, 32, 34]. Where reported, cART regimens were primarily based on protease inhibitors and non-nucleoside reverse transcriptase inhibitors. Further study characteristics are presented in Table 2.

**Table 2:** Intervention and patient characteristics

Study	Country	Start-end date	Total N (I/C)^a^	% with prior HCV therapy	% with concomitant HCV therapy	Age (years)	Male %	Baseline liver damage (%)	Current or past substance abuse (%)
Bruno (2007) [34]	Italy	1999-2004	53 (29/24)	NR	NR	median 37.1	90	100 compensated cirrhosis	NR
Giron-Gonzalez (2007) [29]	Spain	2004-2006	92 (76/22)	19	8	median 40	89	100 compensated cirrhosis	Alcohol: 46 current IDU: 90
Limketkai (2012) [23]	USA	1993-2011	638 (440/198)	NR	NR	median 45.6	66	METAVIR F0-F2: 82 F3: 6 F4: 1	Alcohol: 47 current IDU: 76 past
Macias (2009) [24]	Spain	1986-2008	135 (113/22)	NR	44	mean 37	68	Scheuer F0-F2: 78 F3: 22 F4: 0	Alcohol: 21 current IDU: 90
Macias (2006) [25]	Spain	1991-2005	683 (509/174)	NR	0	median 23^c^	83	Scheuer F0-F2: 68 F3: 18 F4: 14	Alcohol: 23 current IDU: 86 current
Mariné-Barjoan (2004) [35]	France	1997-2000	116 (91/25)	0	0	median 21^c^	67	METAVIR F0-F2: 74 F3-4: 26	Alcohol: 14 current IDU: 72
Mehta (2005) [30]	USA	2001-NR^b^	210 (135/75)	0	NR	median 44.5	67	Ishak F0-F2: 74 ≥F3:26	Alcohol: 39.5 history IDU: 77 current or past
Merchante (2006) [31]	Spain	1997-2004	153 (101/58)	6	NR	median 38	86	100 compensated cirrhosis	Alcohol: 46 current IDU: 88 previous
Pineda (2009) [32]	Spain	1996-2006	154 (145/9)	NR	43	median 39.9	87	100 compensated cirrhosis	Alcohol: 21 IDU: 86 current or previous
Qurishi (2003) [26]	Germany	1990-2002	285 (148/137)	NR	0	median 30	95	No symptomatic liver disease	Alcohol: 12 IDU: 15
Ragni (2009) [27]	USA	1970-2005	85 (60/25)	NR	1	mean 39^d^	100	No cirrhosis	Alcohol: 12 IDU: NR
Reiberger (2010) [36]	Austria	NR-NR	74 (49/25)	0	0	mean 37	77	METAVIR F0-F2: 59 F3-F4: 41	Alcohol: 29 current IDU: NR
Sanmartin (2014) [33]	Spain	1997-2010	162 (149/13)	0	54	mean 37	73	Scheuer F0-F2: 100	Alcohol & IDU: 0 current
Schiavini (2006) [28]	Italy	1985-2002	36 (20/16)	NR	92	median 28	75	Ishak-Knodell F0-2: 75 F3-4: 25	Alcohol: 53 history IDU: NR

ART: 0.279 (0 · 122–0 · 414); untreated: 0.255 (0 · 079–0 · 473); untreated: 145 (SD 43, range 2–610) (time of measurement UC)

^a^I = Intervention, C = Control

^b^Median follow-up 5 years (IQR 2.9-7.5)

^c^At HCV infection

^d^At follow-up

Seven studies reported data on liver-related mortality, [23, 26, 27, 29, 31, 32, 34] and ten studies reported on liver disease progression [24, 25, 27–30, 32, 33, 35, 36]. Three studies reported separate data on both outcomes [27, 29, 32].

### Liver-related mortality

**Fig. 2:**
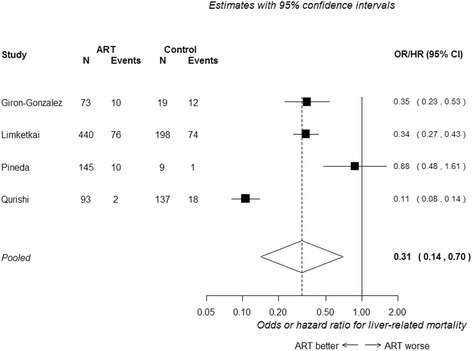
Adjusted odds or hazard of liver-related mortality in HIV/HCV co-infected patients receiving cART versus no cART. Variables adjusted for: Giron-Gonzalez (2007): HCV viral load, liver disease severity, liver disease progression, decompensation during or before follow-up. Limketkai (2012): Age, sex, race, injection drug use, time-varying CD4 cell count and current cART exposure. Pineda (2009): Not reported. Qurishi (2003): Sex, age, risk category, alcohol misuse, HBV, CD4 count, AAT, AST, cholinesterase bilirubin, platelets count, immunoglobulin concentration

Findings from six of the seven studies on liver-related mortality were combined in meta-analyses [23, 26, 27, 29, 32, 34]. Of those, four studies presented analyses adjusted for potential confounding factors [23, 26, 29, 32]. Figure 2 presents a forest plot of the results from these four studies. cART use was associated with a substantial reduction in liver-related mortality, with a hazard/odds around one-third of that in untreated patients (HR/OR 0.31, 95 % CI 0.14 to 0.70). Heterogeneity was high (I^2^ = 95 %), likely to be due to the discordant result between two studies [26, 32]. One showed a much larger benefit; most participants in this study had haemophilia, whereas in the other studies a large majority of patients had a history of IDU.

**Fig. 3:**
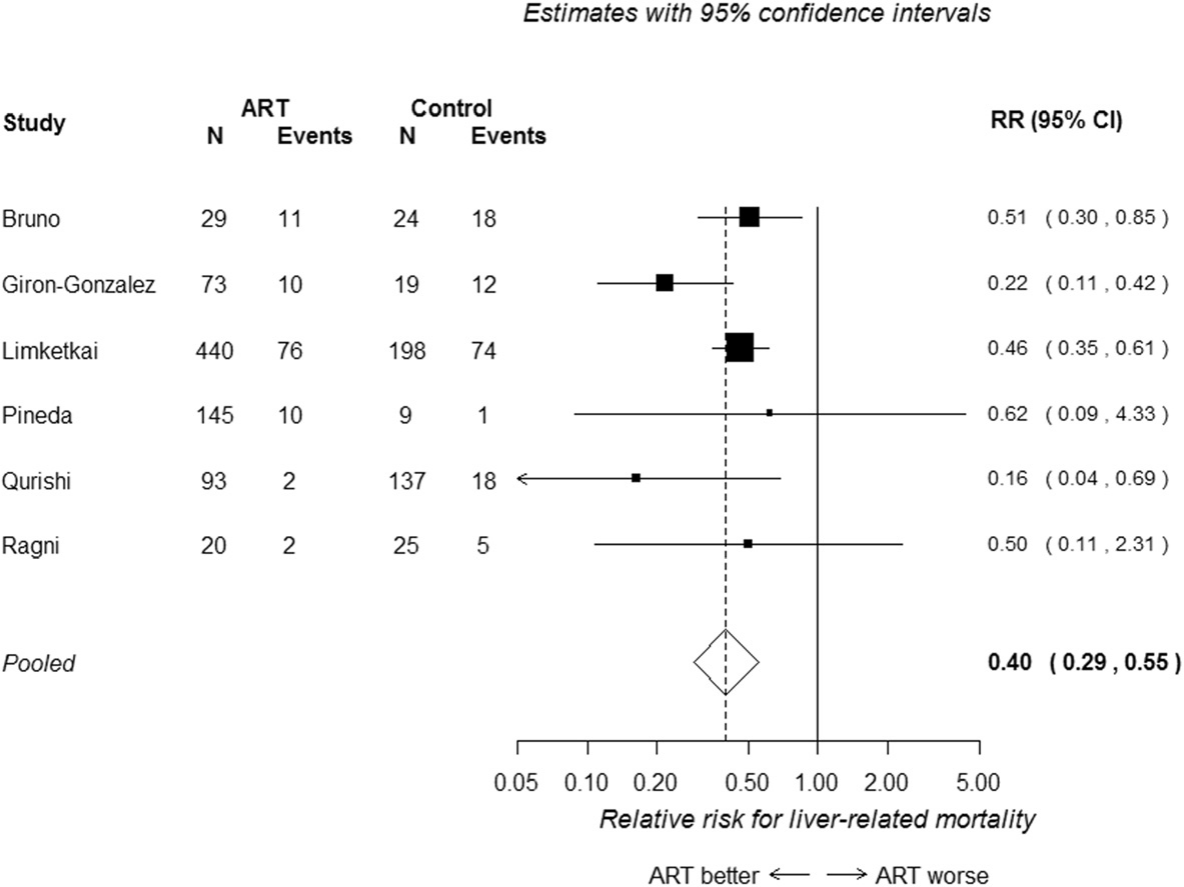
Unadjusted relative risk of liver-related mortality in HIV/HCV co-infected patients receiving cART versus no cART

All six studies included in the meta-analysis presented numbers of patients with and without liver-related mortality from which unadjusted relative risks could be calculated. Figure 3 presents a forest plot of the results. cART is associated with a statistically significant lower risk of liver-related mortality (RR 0.40, 95 % CI 0.29 to 0.55). Moderate heterogeneity was found (I^2^ = 24 %).

### Subgroup analysis

**Fig. 4:**
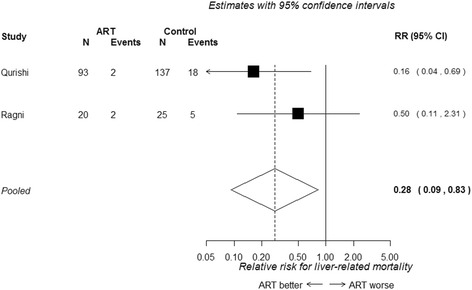
Unadjusted relative risk for liver-related mortality in HIV/HCV co-infected haemophiliac patients receiving cART versus no cART

Figure 4 presents the forest plot for the two studies which included primarily patients with haemophilia. cART is associated with a reduced risk of liver related mortality (RR 0.28, 95 % CI 0.09 to 0.83), but there is too little data to accurately estimate the effect, or to determine if the effect differs from patients with a history of IDU.

### Sensitivity analyses

The number of liver-related deaths per group was not reported in one [23] of the two studies, but it appears that at least 63 % of the events reported across the two study groups were liver-related deaths. Removing this study from the analyses had only a limited effect on the pooled estimates (RR 0.35, 95 % CI 0.21 to 0.57).

Results from one study [31] were not included in the main analyses to avoid the risk of possible double counting with participants from another related study [29]. This study found that the risk of liver-related mortality was significantly lower in cART patients with compensated cirrhosis compared to untreated patients (unadjusted HR 0.5; 95 % CI 0.3 to 0.9). Adding the results of this study to the meta-analysis had a limited effect on the overall findings (RR 0.46, 95 % CI 0.28 to 0.75).

### Liver disease outcomes

Liver disease outcomes were reported too diversely, or in too few studies for meta-analysis and we synthesised the findings narratively [24, 25, 27–30, 32, 33, 35, 36]. A summary of the findings from these studies is presented in Table 3.

**Table 3:** Liver fibrosis progression, decompensation and end stage liver disease outcomes

Study	Intervention	Outcome	Follow-up duration	Effect estimate	Statistically significant?^a^	Adjustments
End-stage liver disease (ESLD) and decompensation events						
Giron-Gonzalez (2007) [29]	cART	Decompensation^j^	Median 20 months (IQR 12 to 28)	HR 0.376 (95 % CI 0.161 to 0.883)	Yes. Favours treatment	Liver disease severity
Giron-Gonzalez (2007) [29]	cART	Decompensation^j^	Median 20 months (IQR 12 to 28)	NR	No	None
Pineda (2009) [32]	cART	Decompensation	Mean 36 months (SD 27), range 1 to 131 months	RR 1.06 (95 % CI 0.30 to 3.71)	No	None
Ragni (2009) [27]	cART & ARV monotherapy	ESLD	NR (up to 35 years from HCV infection)	RR 1.00 (95 % CI 0.37 to 2.71)	No	None
Ragni (2009) [27]	cART vs. ARV monotherapy & no ARV^b^	Time to ESLD	NR (up to 35 years from HCV infection)	HR 3.14 (95 % CI 1.27 to 7.08) (30.3 vs. 20.0 Years)	Yes. Favours treatment	Multivariate (covariates NR)
Liver fibrosis progression (dichotomous)						
Macias (2006) [25]	cART with PI	Liver fibrosis progression^d^	Median 1.6 to 7 years	OR 0.4 (95 % CI 0.2 to 0.7)	Yes. Favours treatment	Age at infection, CD4 count
Macias (2006) [25]	cART with PI switched to efavirenz	Liver fibrosis progression^d^	Median 1.6 to 7 years	OR 0.3 (95 % CI 0.1 to 0.7)	Yes. Favours treatment	Age at infection, CD4 count
Schiavini (2006) [28]	cART and ARV monotherapy	Liver fibrosis progression^e^	Median 54 months (IQR 50 to 86)	OR 2.5 (95 % CI 0.64 to 9.65)	No	None
Macias (2009) [24]	cART and ARV monotherapy	Liver fibrosis progression^h^	3 years	OR 0.94 (95 % CI 0.67 to 1.33)	No	Multivariate^k^
Mehta (2005) [30]	ARV monotherapy	Advanced fibrosis or cirrhosis^c^	Median 5 years (IQR 2.9 to 7.5)	OR 0.61 (95 % CI 0.18 to 2)	No	None
Mehta (2005) [30]	cART	Advanced fibrosis or cirrhosis^c^	Median 5 years (IQR 2.9 to 7.5)	OR 0.92 (95 % CI 0.48 to 1.8)	No	None
Sanmartin (2014) [33]	cART	Liver fibrosis progression^i^	Median 7.8 years (IQR 5.5 to 10.0)	HR 1.94 (95 % CI 0.46 to 8.13)	No	None
Liver fibrosis progression (continuous)						
Macias (2006) [25]	cART with: NVP; or efavirenz; or with PI switched to NVP	Fibrosis progression rate^f^	Median 1.6 to 7 years	Median rate 0.087 to 0.115	No	None
Macias (2006) [25]	cART with zidovudine/lamivudine, or with stavudine/lamivudine	Fibrosis progression rate^f^	Median 1.6 to 7 years	Median rate 0.107 and 0.112	Yes. Favours treatment	None
Mariné-Barjoan (2004) [35]	cART	Fibrosis progression rate^f^	Median 19 to 20 years	MD −0.06 (95 % CI −0.14 to 0.01)	No	None
Reiberger (2010) [36]	cART	Fibrosis progression rate^f^	NR	MD 0.01 (95 % CI −0.01 to 0.04)	No	None
Reiberger (2010) [36]	cART	Time to cirrhosis^g^ from initial HCV exposure	NR	MD −1.00 (95 % CI −7.26 to 5.26)	No	None

*cART* combination antiretroviral therapy, *PI* protease inhibitors, *NVP* nevirapine

^a^ *p* < 0.05

^b^ARV monotherapy patients formed 62 % of the comparator group in this analysis

^c^Ishak score ≥ F3 measured with liver biopsy

^d^Odds of slower fibrosis proression (fibrosis progression rate ≥0.2 vs <0.2), fibrosis measured with liver biopsy

^e^≥1 Knodell-Ishak stage increase between two liver biopsies

^f^METAVIR Fibrosis stage (0 to 3) measured by liver biopsy/length of HCV infection

^g^In years, measured with liver biopsy

^h^≥1 Scheuer stage increase between two liver biopsies spaced by ≥1 year

^i^Liver stiffness value ≥9.5 kPa or died of liver disease

^j^Subgroup without previous decompensation at baseline

^k^Age, undetectable HIV viraemia, genotype 3, ALT and necroinflammatory activity at baseline, time between liver biopsies, HCV treatment response

### End-stage liver disease and decompensation events

Three studies reported data on end-stage liver disease or liver decompensation events [27, 29, 32]. Two of these studies found at least one statistically significant effect in favour of cART [27, 29].

One study [27] found no difference between patients with haemophilia receiving cART and/or ARV monotherapy and untreated patients in the risk of developing end-stage liver disease over 35 years (RR 1.00; 95 % CI 0.37 to 2.71). However, patients receiving cART survived longer before progressing to end-stage liver disease compared with untreated patients (30.3 vs. 20.0 years; HR 3.14; 95 % CI 1.27 to 7.08).

Two studies reported on the risk of liver decompensation in patients with liver cirrhosis [29, 32]. One [29] found a significantly lower risk of a new event of decompensation in cART patients with stable cirrhosis who had experienced decompensation in the past (HR 0.376; 95 % CI 0.161 to 0.883). However, no statistically significant difference was found in the subgroup of patients with no previous decompensation at baseline. Similarly, the other [32] found no significant difference between cART and untreated patients in the risk of decompensation in individuals with cirrhosis.

### Fibrosis progression

Seven studies reported on liver damage in patients with no cirrhosis at baseline, expressed in terms of odds/hazard of fibrosis progression, [24, 25, 28, 30, 33] and/or progression rate [25, 35, 36]. Of the five studies that reported the odds/hazard of fibrosis progression, only one reported a statistically significant difference between intervention and control. This study [25] found significantly lower odds of liver fibrosis progression in patients on cART with protease inhibitors (PI) (OR 0.4; 95 % CI 0.2 to 0.7) and in patients who switched from a PI based regimen to efavirenz during the course of their treatment (OR 0.3; 95 % CI 0.1 to 0.7), but not with other regimens.

Of the three studies that reported fibrosis progression rates, two found a difference in favour of cART [25, 35], and one found no difference between cART and no treatment [36]. One [25] found slower median rates of fibrosis progression in patients treated with cART compared with untreated patients, regardless of regimens used. However, the difference was only statistically significant for some regimens (zidovudine/lamivudine and stavudine/lamivudine). Another [35] found a slower mean rate of fibrosis progression over approximately 15 years in patients undertaking cART at follow-up, although the difference did not reach statistical significance.

## Discussion

We included 14 studies evaluating the association between cART and/or ARV monotherapy and liver disease progression and liver-related mortality in patients co-infected with HIV and hepatitis C. In most studies the majority of patients had a history of IDU, except for two studies that included only or mostly participants with haemophilia. cART was found to be associated with a substantial reduction in liver-related mortality, with a chance/hazard around one-third of that in untreated patients. Pooled estimates from unadjusted analyses also showed a clear association in favour of cART for preventing liver-related mortality. A subgroup analysis including nearly all patients with haemophilia also found a reduced incidence of liver related mortality in individuals receiving cART, but there were too little data to provide an accurate estimate or to determine if the effect differed from other populations. Findings for other liver-related outcomes were less consistent, although no studies reported that lack of cART or ARV monotherapy was associated with significantly better liver-disease outcomes.

### Strengths and limitations

This systematic review was conducted following the general principles recommended in CRD Guidance for Undertaking Reviews in Health Care, and the reporting guidance of the PRISMA and MOOSE statements [15, 16]. Study quality was assessed systematically and considered when interpreting the findings. Whenever possible, data on treatment effect for individual studies were extracted or calculated, even when quantitative synthesis was not undertaken. The review was completed within a ten-week timeframe to meet the needs of the Department of Health in England and due to time constraints we did not search for conference abstracts, included only English language studies and one reviewer screened titles and abstracts. This means that relevant studies may have been missed, and the risk of publication bias cannot be ruled out. Quantitative assessment of publication bias was considered inappropriate due to the limited number of included studies. Despite the limitations of our searches, we believe it is unlikely that any potential missed studies would significantly modify the findings of the main analyses on liver-related mortality and our main conclusions. This is because the observed effect associated with cART is substantial.

Findings from most studies on liver-related mortality were synthesised quantitatively. Adjusted and unadjusted results were pooled separately as an attempt to address potential confounding. Adjusted mortality values could only be combined based on the (inaccurate) assumption that odds and hazard ratios are equivalent, and this approach could create heterogeneity across studies because of different analysis methods used to obtain the adjusted results; therefore these results need to be interpreted with caution. However, the pooled estimates from adjusted and unadjusted values did not differ significantly, and both suggested substantial benefits of cART.

Liver disease outcomes were reported too diversely, or in too few studies for statistical pooling. This limits the strength of the findings on liver disease progression. There were too few studies to conduct meta-regression or further subgroup analyses to explore the moderating effect of several relevant factors, including age, liver disease severity, baseline CD4 count, HBV co-infection, co-intervention with HCV therapy, time since HCV/HIV infection, HIV treatment duration treatment history of HCV infection or alcohol abuse.

Unsurprisingly, no RCTs were identified and all included studies were observational. Given the known overall survival benefits associated with cART, it would be unethical to randomise patients to no cART. Half of the studies adjusted for potential confounders such as age or sex, although the variables accounted for varied across the studies. For instance, only two studies controlled for alcohol misuse in their analyses. Although attempts were made to address the risk of confounding in the analyses, risk of confounding cannot be ruled out. The pooled analyses showed heterogeneity, particularly for the meta-analysis of adjusted results, which limits the strength of the review findings.

Studies might have been affected by a survivorship bias if patients in the intervention group who survived long enough to receive treatment had slower HCV progression, and therefore may have had better HCV-related outcomes [13]. The use of a time-dependent variable or Cox proportional hazards modelling taking HCV duration or progression into account might have remedied this bias. However, no studies reported using this technique. On the other hand, it is possible that comparison groups had levels of immunosuppression that were considered sufficiently high for their treatment to be delayed [37–39]. In this case, patients in the ART group may have had poorer health at treatment initiation, and may therefore have been at higher risk of liver disease progression. Unfortunately, reporting of participant characteristics in the studies was insufficient to support or reject these assumptions.

Reasons for not receiving cART or ARV monotherapy were generally not reported. However, given that cART, and previously ARV monotherapy, would likely be recommended to most HIV/HCV co-infected individuals, particularly those with high HIV viral load, reasons for not receiving treatment were likely influenced by individual patient choice. Those receiving cART may be less likely to be active IDUs (for example, ex-IDUs on methadone programmes) and may have different lifestyles (for example, less alcohol and substance abuse) compared to those who do not receive cART. Reporting of baseline differences between cART/ARV monotherapy and untreated groups was often limited. Although no studies reported significant differences between groups such as current alcohol, IDU or other substance abuse, and although some studies adjusted for these variables in their analyses, it is still possible that those who received treatment for HIV were different to those who did not for reasons that may have influenced liver-related outcomes.

Where reported, most participants had a history of IDU. This should be taken into account when interpreting the results of the review. Most participants included in the studies were under 50 years of age and the burden of other co-morbidities is likely to be higher in older populations. This, in addition to the toxicity of other treatments, may impact differently upon liver disease. This limits the applicability of the findings to older populations, especially given the increasing life expectancy of people with HIV and HCV, and the growing proportion of people with HIV aged 50 years and over.

We identified only studies from high-income countries and note that the applicability of the review findings to low- and middle-income countries is uncertain.

### Implications for policy, practice and further research

This systematic review provides an up-to-date synthesis of the available evidence on the effect of cART and ARV monotherapy on liver disease progression and liver-related mortality in individuals co-infected with HIV and hepatitis C. This review, together with another review on quality of life and extrahepatic conditions in individuals with chronic hepatitis C, [40] was commissioned as part of ongoing policy consideration about the shape of support for those affected by hepatitis C or HIV from historic NHS blood treatments before donor screening tests or virus inactivation methods were available in the UK. A public consultation on reform of the existing financial and other support available was announced in January 2016.

The findings of this review support the use of cART in patients co-infected with HIV and HCV as recommended by current guidelines [39, 41]. Given the increased risk of liver-related morbidity and mortality in patients co-infected with HIV and HCV and the limited evidence on the impact of cART on liver disease progression, the need for monitoring liver-disease progression in this population clearly remains. Future management of patients co-infected with HIV and HCV is likely to evolve with the advent of new directly acting antivirals (DAAs) for the treatment of HCV, [42–45] and recent trials have found high sustained virologic response (SVR) rates in non-cirrhotic patients co-infected with HIV and HCV with certain DAA combinations [46, 47].

Few included studies reported data separately for different antiretroviral classes and combinations. Several studies comparing different ARV regimens did not compare cART and/or ARV monotherapy with no HIV treatment and were therefore excluded from our review. Given the ubiquitous use of cART in HIV management, a systematic review on the acute and chronic effect of different cART regimens would be relevant. The mechanisms by which liver disease mortality is reduced with cART are still largely unknown [27]. Further research would clarify whether the effect of cART on liver-disease progression and mortality may occur through immune reconstitution, viral suppression or a combination of both [13].

## Conclusions

The use of cART was found to be associated with a significantly reduced risk of liver-related mortality in patients co-infected with HIV and HCV. Evidence of a positive association with liver disease progression is less clear, although there is no evidence to suggest that the absence of cART and/or ARV monotherapy is preferable.

## Additional files

Additional file 1: Search strategy. (DOCX 14 kb)

Additional file 2: Quality assessment and risk of bias. (DOCX 17 kb)

## Abbreviations

ARV: Antiretroviral therapy
cART: Combination antiretroviral therapy
HCV: Hepatitis C virus
HR: Hazard ratio
IDU: Injection drug use
MD: Mean difference
OR: Odds ratio
RR: Relative risk
