# Supplementary material for: Antiretroviral therapy and liver disease progression in HIV and hepatitis C co-infected patients: a systematic review and meta-analysis
Source: Hepatol Med Policy. 2016 Aug 15;1:10. doi: 10.1186/s41124-016-0015-7 (PMC5918754; doi:10.1186/s41124-016-0015-7)
Supplement: Supplementary file 1 — Search strategy. (DOCX 14 kb) [file 41124_2016_15_MOESM1_ESM.docx]

## S.1 MEDLINE Search Strategy

**MEDLINE**
Searched 18/06/14 and updated 09/09/2015 via OVID interface.

Database: Ovid MEDLINE(R) In-Process & Other Non-Indexed Citations and Ovid MEDLINE(R) <1946 to Present>
No date or language limits applied.

Search Strategy:

1 exp HIV/ (84307)

2 exp HIV Infections/ (233038)

3 exp Hepatitis C/ (47865)

4 exp Hepacivirus/ (24124)

5 (1 or 2) and (3 or 4) (7098)

6 exp Antiretroviral Therapy, Highly Active/ (17442)

7 Anti-Retroviral Agents/ (5840)

8 Antiviral Agents/ (56460)

9 Anti-HIV Agents/ (34699)

10 6 or 7 or 8 or 9 (106310)

11 5 and 10 (2269)

12 exp Fibrosis/ (50801)

13 Liver Cirrhosis/ (56237)

14 Liver Diseases/ (58223)

15 Liver/ (373600)

16 Drug-Induced Liver Injury/ (23066)

17 (liver adj2 (fibrosis or cirrhosis)).ti,ab. (29669)

18 12 or 13 or 14 or 15 or 16 or 17 (530439)

19 11 and 18 (559)

Numbers presented are from 18/06/2014 search

The EMBASE strategy was developed using a similar template and is available on request.
