# Supplementary material for: Antiretroviral therapy and liver disease progression in HIV and hepatitis C co-infected patients: a systematic review and meta-analysis
Source: Hepatol Med Policy. 2016 Aug 15;1:10. doi: 10.1186/s41124-016-0015-7 (PMC5918754; doi:10.1186/s41124-016-0015-7)
Supplement: Supplementary file 2 — Quality assessment and risk of bias. (DOCX 17 kb) [file 41124_2016_15_MOESM2_ESM.docx]

## S.2 Quality assessment and risk of bias

Risk of bias was evaluated using a modified version of the Newcastle-Ottawa quality assessment tool.[20] As with the Newcastle-Ottawa tool, three main domains were considered: participant selection, confounding, and outcomes. However, questions were devised to inform a judgment about risk of bias for each of these three domains within each study, whereas the Newcastle-Ottawa tool is designed to provide an overall quality score to each study. We felt that the use of quality scores was potentially misleading. For instance, a study with high risk of confounding due to lack of adjustment in its analysis may still receive a relatively high quality score if it meets all other criteria. Questions informing each quality criteria are presented below.

S.2 Table 1 Quality assessment criteria

| **Participant selection** | |
| --- | --- |
| Question 1 | Was the sample representative of study population of interest? (Yes/No/Unclear) |
| Question 2 | Was there no presence of outcome at baseline? (Yes/No/Unclear) |
| Question 3 | Was HCV assessed using valid methods (e.g. polymerase chain reaction)? (Yes/No/Unclear) |
| Risk of selection bias | Risk of bias associated with participant selection (High/Low/Unclear) |
| **Confounding** | |
| Question 4 | Group differences: Were there significant differences in population characteristics between intervention and comparison group? (Yes/No/Unclear) |
| Question 5 | Was there adjustment for relevant confounders in the analyses? (e.g. age, sex/gender, duration of HCV infection, alcohol abuse, HCV treatment, baseline liver damage) (Yes/No/Unclear) |
| Question 6 | Was risk of cross-over addressed? (i.e. risk that comparison group received ART during the study) (Yes/No/Partly) |
| Risk of confounding bias | Risk of bias associated with confounding (High/Low/Unclear/Moderate) |
| **Outcome measurement** | |
| Question 7 | Was the outcome assessed using valid methods? (e.g. using hospital registry data) (Yes/No/Unclear) |
| Question 8 | Was follow-up duration adequate/was follow-up sufficient to assess the impact of the intervention on the outcome of interest? (report assessment separately if the answer differs per outcome) (Yes/No/Unclear) |
| Question 9 | Was loss to follow-up acceptable? (Yes/No/Unclear/Not applicable) |
| Risk of outcome measurement bias | Risk of bias associated with outcome measurement (High/Low/Unclear) |
